# Corneal Epithelial Remodeling and Its Effect on Corneal Asphericity after Transepithelial Photorefractive Keratectomy for Myopia

**DOI:** 10.1155/2016/8582362

**Published:** 2016-09-08

**Authors:** Jie Hou, Yan Wang, Yulin Lei, Xiuyun Zheng, Ying Zhang

**Affiliations:** ^1^Clinical College of Ophthalmology, Tianjin Medical University, Tianjin 300020, China; ^2^Tianjin Eye Hospital, Tianjin 300020, China; ^3^Tianjin Key Lab of Ophthalmology and Visual Science, Tianjin 300020, China; ^4^Tianjin Eye Institute, Tianjin 300020, China; ^5^Jinan Mingshui Eye Hospital, Zhangqiu, Shandong 250200, China

## Abstract

*Purpose.* To evaluate the changes in epithelial thickness profile following transepithelial photorefractive keratectomy (T-PRK) for myopia and to investigate the effect of epithelial remodeling on corneal asphericity.* Methods.* Forty-four patients (44 right eyes) who underwent T-PRK were retrospectively evaluated. Epithelial thickness was measured using spectral-domain optical coherence tomography at different corneal zones (central, 2 mm; paracentral, 2–5 mm; and mid-peripheral, 5-6 mm) preoperatively and at 1 week and 1, 3, and 6 months postoperatively. The correlation between the changes in corneal epithelial thickness (ΔCET) and postoperative *Q*-value changes (ΔQ) was analyzed 6 months postoperatively.* Results.* Epithelial thickness at 6 months showed a negative meniscus-like lenticular pattern with less central thickening, which increased progressively toward the mid-periphery (3.69 ± 4.2, 5.19 ± 3.8, and 6.23 ± 3.9 *μ*m at the center, paracenter, and mid-periphery, resp., *P* < 0.01). A significant positive relationship was observed between epithelial thickening and ΔQ 6 months postoperatively (*r* = 0.438, 0.580, and 0.504, resp., *P* < 0.01).* Conclusions.* Significant epithelial thickening was observed after T-PRK and showed a lenticular change with more thickening mid-peripherally, resulting in increased oblateness postoperatively. Epithelial remodeling may modify the epithelial thickness profile after surface ablation refractive surgery for myopia.

## 1. Introduction

In recent years, owing to refractive accuracy and stability of excimer laser ablation concerns, research on epithelial thickness profile changes after myopic surgery has gained general interest [1, 2]. The majority of studies have noted that epithelial thickness increased after myopic procedures; however, these studies were only based on central epithelial thickness measurements. The thickness distribution in normal eyes is uneven from the center of the cornea to the periphery [3–5]. Nonuniformity in the thickness of corneal epithelium has also been observed after laser in situ keratomileusis (LASIK) [6, 7] and photorefractive keratectomy (PRK) [8].

Corneal surface ablation such as PRK has been the better choice for eyes with thin corneas because it preserves more corneal biomechanical properties than LASIK [9, 10]. However, associated pain, irregular epithelial healing, and corneal haze are the drawbacks of this procedure. New advanced surface ablation techniques, such as transepithelial photorefractive keratectomy (T-PRK) [11], have been developed to reduce these complications.

Until now, changes in the full-corneal epithelial thickness profile after T-PRK and the effect of epithelial remodeling on corneal asphericity in accordance with clinical data are not well described. In this study, we evaluate the postoperative changes in epithelial thickness centrally, paracentrally, and mid-peripherally after T-PRK by using high-resolution spectral-domain optical coherence tomography (SD-OCT) and to investigate the potential association of these changes with *Q*-value change.

## 2. Materials and Methods

### 2.1. Patients

Forty-four myopic patients (right eyes) who had received treatment for T-PRK were enrolled from August 2014 to March 2015 in this retrospective study. The study protocol was approved by the ethics committee and adhered to the tenets of the Declaration of Helsinki. The inclusion criteria were age ≥18 years, no ocular disease or systemic disease, no epithelial defects, no dry eye disorder, spherical equivalent (SE) refraction ≤−6.00 diopters (D), refractive diopter maintained stable for more than 2 years, preoperative corneal central thickness (CCT) >460 *μ*m as measured by OCT, and discontinuation of soft contact lens wear or rigid gas-permeable contact lens (RGP) at least 2 weeks or more than 1 month before surgery, respectively.

All patients underwent preoperative and postoperative examinations including uncorrected visual acuity, best-corrected visual acuity, manifest and cycloplegic refractions, noncontact intraocular pressure, anterior segment slit-lamp microscopy, and corneal topography with the Scheimpflug tomography system (Pentacam; Oculus GmbH, Wetzlar, Germany). The *Q*-value of the cornea within the 6 mm corneal diameter was assessed using the Pentacam before surgery and 6 months postoperatively. Patients were followed up at 1 week and 1, 3, and 6 months postoperatively.

### 2.2. Epithelial Thickness Measurements

The RTVue Fourier-domain OCT system (v. 6.11.0.12, Optovue Inc., Fremont, CA, USA) was used to measure epithelial thickness and corneal thickness. The device has a scan speed of 26000 axial scans per second using a wavelength of 830 nm. A “Pachymetry + Cpwr” scan pattern (6 mm scan diameter, eight radials, 1024 axial scans each, repeated five times) centered on the pupil was used to map the cornea. The epithelial thickness map was generated by an automatic algorithm and divided into a total of 17 sectors: (1) a central 2 mm diameter zone, (2) eight paracentral octants between 2- and 5 mm diameter rings, and (3) eight mid-peripheral octants between 5- and 6 mm diameter rings (Figure 2(a)). For each of these sectors, average thickness was displayed over the corresponding area. Each eye was scanned three times and the average value was used for further analysis.

### 2.3. Surgical Technique

The same surgeon performed all surgeries (Jie Hou). Preoperatively, each eye received one drop of proparacaine (Alcaine), a sterile drape was applied, and a lid speculum was inserted.

The epithelium and stroma were ablated in a single step using the aberration-free transepithelial PRK mode of the Schwind Amaris 750 Hz (Schwind Eye-tech-solutions GmbH, Kleinostheim, Germany). The module integrates aspheric ablation profiles that compensate for the peripheral loss of energy caused by an increased angle of incidence on the cornea. For each treatment, the optical zone (OZ) diameter varied between 6.30 and 7.10 mm based on the pupil diameter and the type of refractive error. In consideration of the potential toxic influence on corneal tissue, mitomycin was not used in those patients whose average SE refraction of the subjects was ≤−6.00 D.

After photoablation, the cornea was irrigated with a cool balanced salt solution. A soft bandage contact lens was applied for 3 to 4 days. All patients were instructed to use 0.5% levofloxacin (Cravit; Santen, Inc.) four times a day for 1 week, and 0.1% fluorometholone (Allergan, Inc.) drops were initiated four times a day after epithelial closure and contact lens removal; the drops were tapered progressively over the following 4 months.

### 2.4. Statistical Analysis

Statistical analyses were performed using IBM SPSS Statistics, version 20 (IBM Corp., Armonk, NY, USA). The average value of the eight octants within the different annulus between the circles was calculated as the paracentral or mid-peripheral thickness. All changes were calculated as follows: 6-month postoperative − preoperative value; in this paper, changes are preceded by the Δ symbol. The distributions of variables were determined using Kolmogorov-Smirnov tests. The mean and standard deviation were used for descriptive statistics. Mean epithelial thicknesses at different examination points and different measurement zones were compared by repeated-measures analysis of variance. Postoperative changes in corneal epithelial thickness (ΔCET) superiorly versus inferiorly and nasally versus temporally were calculated using the paired *t*-test. The Pearson correlation coefficient (*r*) was used to evaluate correlations between variables while multiple linear regression analysis was used to explore factors influencing ΔCET. A *P* value less than 0.05 was considered statistically significant.

## 3. Results

The mean age of all patients at the time of surgery was 21.70 ± 5.1 years. The mean SE refraction was −3.96 ± 1.3 D and the mean preoperative central corneal thickness measured by OCT was 482.16 ± 40.7 *μ*m. The mean OZ diameter was 6.52 ± 0.2 mm, while the ablation depth, including the epithelium, was 117.67 ± 19.9 *μ*m. Mean corneal epithelial thickness at the center, paracenter, and mid-periphery was 52.78 ± 3.3, 52.83 ± 3.0, and 52.47 ± 2.7 *μ*m, respectively.

### 3.1. Changes in Postoperative Epithelial Thickness

**Figure 1:**
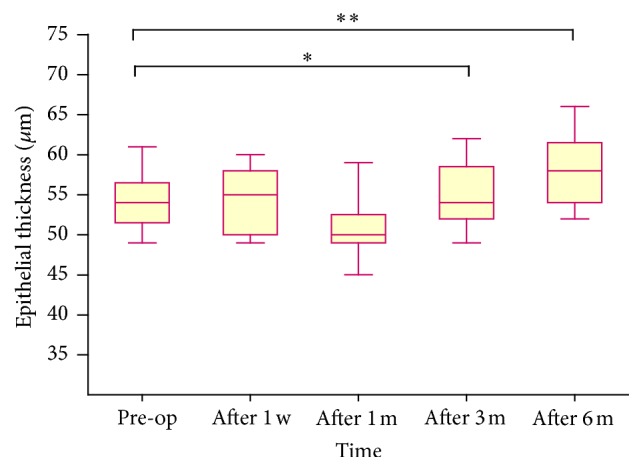
Change in central corneal epithelial thickness over time after T-PRK. The epithelium was significantly thicker at 3 and 6 months after surgery compared with preoperative measurements. ^*∗∗*^ *P* < 0.001, ^*∗*^ *P* < 0.05.

Table 1 shows the epithelial thickness at increasing radial distances from the corneal vertex over time. A slight increase in central epithelial thickness was observed at 1 week and a slight decrease at 1 month postoperatively. However, no significant differences were found between the two follow-up points (*P* = 0.261 and *P* = 0.297, resp.). The epithelium was significantly thicker at 3 and 6 months postoperatively compared with preoperative measurements (*P* = 0.027 and *P* < 0.001, resp.) (Figure 1). After annular averaging, this trend was also observed at the paracentral and mid-peripheral zones (Table 1).

**Figure 2:**
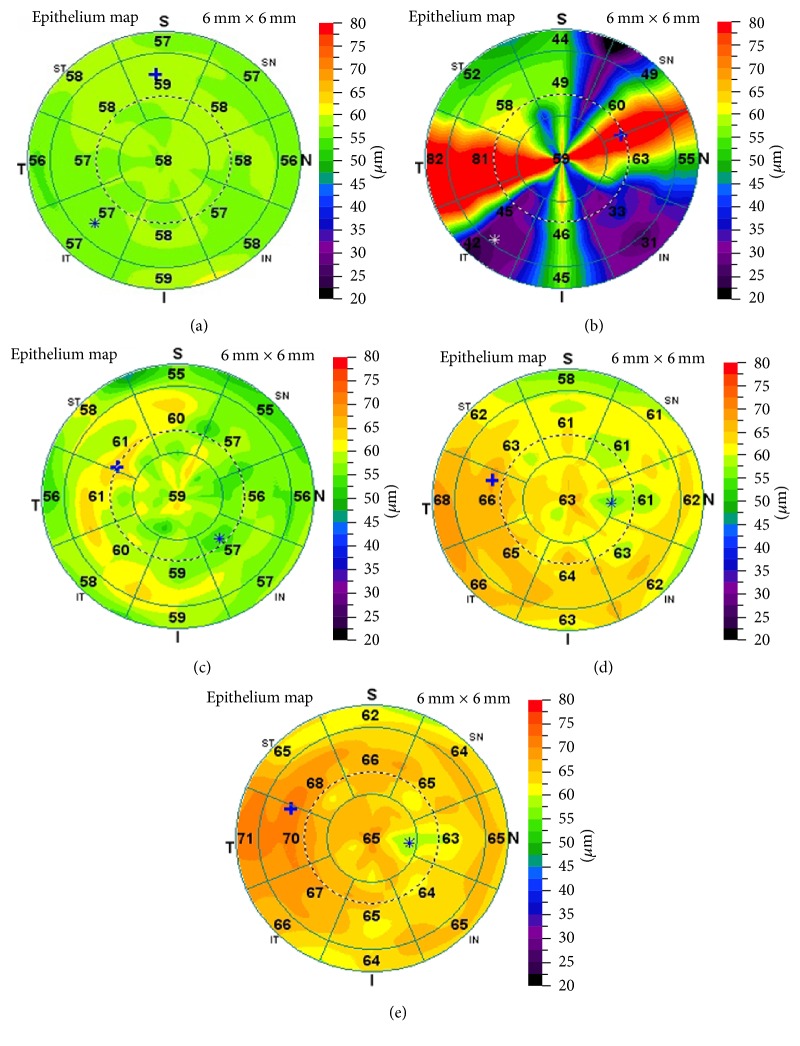
Epithelial thickness profile mapping seen preoperatively (a) and 1 week (b), 1 month (c), 3 months (d), and 6 months (e) postoperatively in a T-PRK examination. The patient (right eye) received treatment for −4.75 D.

**Figure 3:**
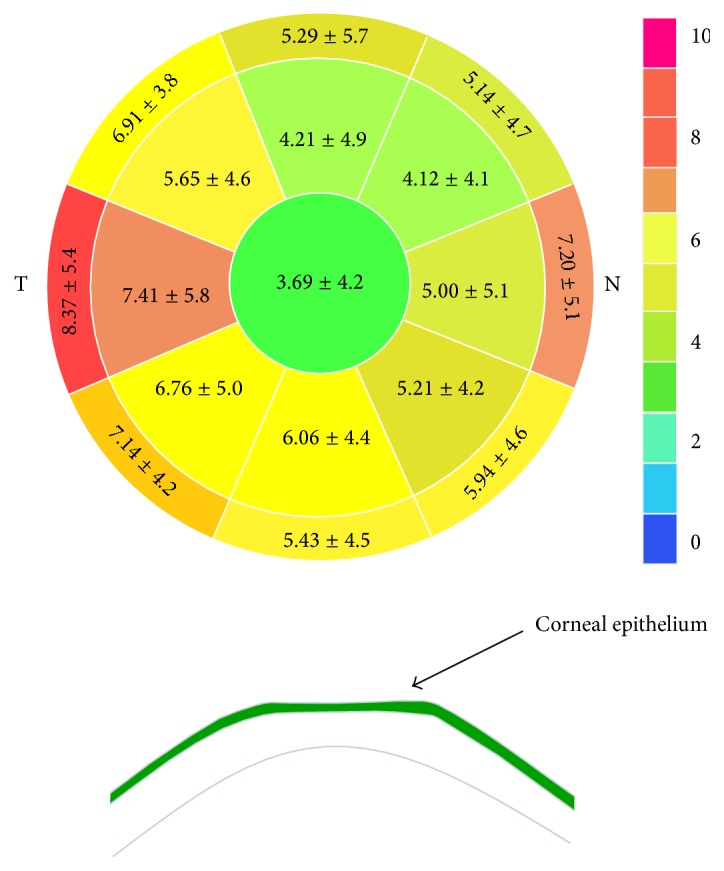
Epithelial thickening at each sector 6 months postoperatively (mean ± standard deviation, *μ*m).

At 6 months postoperatively, the epithelial thickness showed a thickening of 3.69 ± 4.2, 5.19 ± 3.8, and 6.23 ± 3.9 *μ*m at the central, paracentral, and mid-peripheral zones, respectively (*P* < 0.001). A significant change in epithelial thickness was detected among the three zones (*P* = 0.030), and the most pronounced change was observed at the mid-periphery zone (*P* = 0.008). After the same sector averaging, the epithelium was 1.67 ± 2.07 *μ*m thinner superiorly than inferiorly (*P* < 0.001) and 1.00 ± 1.00 *μ*m thinner nasally than temporally (*P* = 0.002). The maximum amount of epithelial thickening was 8.37 ± 5.4 *μ*m at the temporal zone (Figures 2 and 3).

### 3.2. Correlation and Regression Analysis

**Figure 4:**
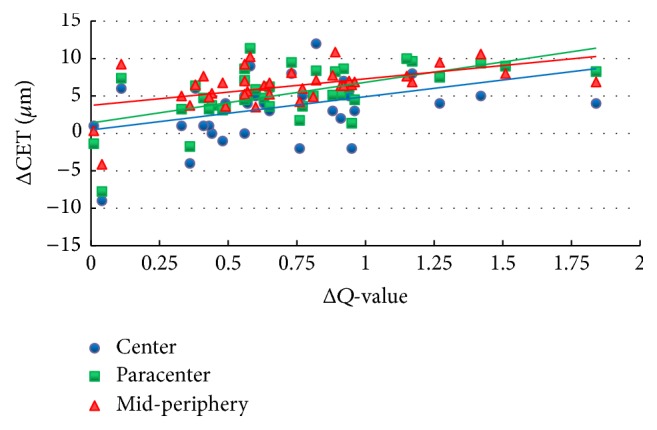
Correlation between ΔCET and Δ*Q*-value 6 months postoperatively.

Table 2 shows the *r*-values and associated *P* values for the analysis of the relationship between ΔCET and Δ*Q*-value, between ΔCET and SE, between ΔCET and ablation depth, and between ΔCET and OZ diameter at 6 months postoperatively. The anterior corneal surfaces showed an oblate shift within the ablation zone. The *Q*-value increased to 0.73 ± 0.39 in eyes undergoing T-PRK.  Figure 4 shows that the epithelial thickening at each zone was positively correlated with ΔQ (*P* < 0.01). There was also a statistically significant correlation between ΔCET and SE. Epithelial thickening was correlated with ablation depth paracentrally and mid-peripherally (*r* = 0.380 and 0.383; *P* < 0.05). Epithelial thickening at each zone was negatively correlated with OZ diameter (*P* < 0.01).

Multiple linear regression models were constructed to explore factors influencing ΔCET 6 months postoperatively. Optical zone diameter and Δ*Q* were enrolled into regression equations. The regression equations were as follows: ΔCET_0−2 mm_ = 0.278 + 4.844Δ*Q* (*R* ^2^ = 0.185, *F* = 7.933, *P* = 0.008); ΔCET_2−5 mm_ = 42.349 + 3.775Δ*Q* − 6.134OZ (*R* ^2^ = 0.408, *F* = 11.719, *P* = 0.000); and ΔCET_5-6 mm_ = 62.122 + 3.739Δ*Q* − 9.019OZ (*R* ^2^ = 0.426, *F* = 5.940, *P* = 0.001).

## 4. Discussion

The corneal epithelium has the ability to alter its thickness profile to reduce surface irregularities and to reestablish a smooth optical surface [12]. Histological studies have shown that these epithelial changes include elongation of the basal epithelial cell layer and an increase in the number of superficial cell layers [2]. Such compensatory changes in the corneal epithelium after myopic excimer laser ablation have been demonstrated using Artemis very high frequency digital ultrasound [6], confocal microscopy [2], and OCT [4, 5] in several previous studies. All of these studies showed that increased epithelial thickness after excimer laser surgery is caused by epithelial hyperplasia. The hyperplasia not only is a response to epithelial removal during PRK but also occurs after LASIK, during which the central epithelium remains intact [1, 2]. Furthermore, a previous study speculated that the change in anterior corneal contour after laser ablation stimulates epithelial thickening [2].

The reliability, reproducibility, and axial resolution of corneal epithelial thickness measurements by SD OCT have already been reported [3, 13, 14]. Rocha et al. [5] and Chen et al. [8] applied this technique to acquire epithelial thickness profiles after LASIK and PRK. To the best of our knowledge, no previous study has used SD-OCT to evaluate the role of the epithelial remodeling on the anterior corneal asphericity after corneal refractive surgery.

In the present study, the epithelial debridement in the surface ablation caused an initial edema and nonuniform thickening in central corneal epithelium 1 week postoperatively, and then epithelial thickness was reduced after 1 month, followed by a gradual epithelial thickening over the following 6 months. The mechanism underlying the thinning of the corneal epithelium at 1 month postoperatively is not well understood. However the sensory nerves transection and reduction of trophic modulator secretion after laser ablation may explain the thinning of the corneal epithelium [15, 16]. The regeneration of subepithelial corneal nerves is usually active 3 months postoperatively [17]. In our study, increased thickening of the corneal epithelium was observed in the third postoperative month, further indicating that the corneal nerve regeneration may play an important role in postoperative epithelial remodeling. The duration of epithelial thickening was longer than that reported in a previous study on LASIK (1 week) [1, 18], possibly owing to differences in initial epithelial wound healing between laser surface ablation and LASIK.

The nonuniform preoperative epithelial thickness profile has been reported to be thicker inferiorly and nasally in normal eyes [2, 4, 8]. We also found that the epithelium thickening was characterized by a thicker epithelium inferiorly than superiorly and temporally than nasally after T-PRK, with the maximum amount of epithelial thickening observed temporally, in accordance with the findings of Chen et al. on PRK [8].

Furthermore, a negative meniscus-like lenticular change in the epithelial thickness profile, with less thickening centrally and a progressively increasing thickening toward the periphery, was observed after T-PRK in the current study. The epithelial thickness at the central, paracentral, and mid-peripheral zones was 3.69 ± 4.2, 5.19 ± 3.8, and 6.23 ± 3.9 *μ*m, respectively, at 6 months postoperatively, with a 2.5-*μ*m thicker epithelium mid-peripherally than centrally. This pattern is consistent with the post-PRK epithelial thickness profile changes noted by Chen et al. [8] using the same measurement technology but in contrast to the post-LASIK epithelial thickening detected using Artemis, which showed more thickening centrally and progressively less thickening toward the periphery [6]. This might be explained by the aspheric ablation profile in the Amaris results, with increased peripheral tissue ablation compared with the center of the cornea. Therefore, the corneal epithelium may compensate for this transition zone by having a reduced thickness centrally. This was confirmed by the statistical relationship between ablation depth and paracentral epithelial thickening after surgery in the present study.

It is widely acknowledged that myopic laser ablation changes the cornea from prolate to oblate shape [19–21]. Kwon and Bott [22] and Huang et al. [23] applied a mathematical model to explain the origin of changes in corneal asphericity after laser refractive surgery. They showed that corneal remodeling changes the corneal shape after surgery by making the center of the cornea flatter and the periphery steeper, resulting in a more oblate cornea.

An additional finding of our study was that the lenticular epithelial thickening had a significant positive effect on the corneal asphericity, indicating that the differential increase in epithelial thickness profile could account for the observed clinical trend to oblateness. The thicker the epithelial thickness, the more pronounced the shift. In addition, we explored the positive correlation between ΔCET and SE and between ΔCET and ablation depth. This emphasizes that greater attention should be paid to epithelial remodeling after corneal refractive surgery, especially for high myopic patients with aspheric profiles of ablation in the correction of spherical aberration. We also found a significant negative correlation between postoperative epithelial thickening and OZ diameter, indicating that epithelial hyperplasia was greater with smaller zone sizes. This is in agreement with previous findings [6, 8].

One limitation of the current study is that the results are not representative of the general myopic eye population because some of our patients had corneas that were slightly thinner than the average. Another limitation is the inability of SD-OCT to measure epithelial thickness outside the 6 mm diameter of the cornea.

In summary, the present study demonstrated that a specific remodeling occurred from the center to the periphery of the cornea after T-PRK for myopia. The lenticular change in the epithelial thickness profile after T-PRK may be caused by more peripheral tissue ablation. This change results in increased oblateness and may counterbalance the positive effect of restoring the prolate shape of the central cornea. Further investigations are required to evaluate the long-term effect of corneal epithelial changes and the potential effect on visual quality.

## Figures and Tables

**Table 1:** Epithelial thickness profile at 1 week, 1 month, 3 months, and 6 months after T-PRK.

CET (*μ*m)	1 week			1 month		
	Central	Para	Mid	Central	Para	Mid
Mean ± SD	53.97 ± 4.3	53.50 ± 4.3	51.94 ± 5.5	51.03 ± 4.1	52.96 ± 3.6	53.68 ± 3.4
*P*	*P* = 0.261	*P* = 0.487	*P* = 0.192	*P* = 0.297	*P* = 0.894	*P *= 0.192

CET (*μ*m)	3 months			6 months		
	Central	Para	Mid	Central	Para	Mid

Mean ± SD	55.14 ± 5.5	56.61 ± 4.8	57.18 ± 4.1	56.68 ± 5.1	58.03 ± 4.6	58.70 ± 3.7
*P*	*P* = 0.027	*P* < 0.001	*P* < 0.001	*P* < 0.001	*P* < 0.001	*P *< 0.001

*P* value in comparison with preoperative measurements; SD: standard deviation.

**Table 2:** The correlation between ΔCET and Δ*Q*-value, SE, and AD 6 months postoperatively.

Parameter	*r*-value (*P* value)			
	Δ*Q*-value	SE	AD	OZ
Central	0.438 (0.007)	0.380 (0.020)	0.216 (0.199)	−0.427 (0.008)
Para	0.580 (<0.001)	0.492 (0.002)	0.380 (0.020)	−0.554 (<0.001)
Mid	0.504 (0.001)	0.423 (0.009)	0.383 (0.019)	−0.557 (<0.001)

## References

[1] Ivarsen A., Fledelius W., Hjortdal J. Ø. (2009). Three-year changes in epithelial and stromal thickness after prk or lasik for high myopia. *Investigative Ophthalmology and Visual Science*.

[2] Patel S. V., Erie J. C., McLaren J. W., Bourne W. M. (2007). Confocal microscopy changes in epithelial and stromal thickness up to 7 years after LASIK and photorefractive keratectomy for myopia. *Journal of Refractive Surgery*.

[3] Kanellopoulos A. J., Asimellis G. (2013). In vivo three-dimensional corneal epithelium imaging in normal eyes by anterior-segment optical coherence tomography: a clinical reference study. *Cornea*.

[4] Li Y., Tan O., Brass R., Weiss J. L., Huang D. (2012). Corneal epithelial thickness mapping by fourier-domain optical coherence tomography in normal and keratoconic eyes. *Ophthalmology*.

[5] Rocha K. M., Perez-Straziota C. E., Stulting R. D., Randleman J. B. (2013). SD-OCT analysis of regional epithelial thickness profiles in keratoconus, postoperative corneal ectasia, and normal eyes. *Journal of Refractive Surgery*.

[6] Reinstein D. Z., Archer T. J., Gobbe M. (2012). Change in epithelial thickness profile 24 hours and longitudinally for 1 year after myopic LASIK: three-dimensional display with artemis very high-frequency digital ultrasound. *Journal of Refractive Surgery*.

[7] Rocha K. M., Krueger R. R. (2014). Spectral-domain optical coherence tomography epithelial and flap thickness mapping in femtosecond laser-assisted in situ keratomileusis. *American Journal of Ophthalmology*.

[8] Chen X., Stojanovic A., Liu Y., Chen Y., Zhou Y., Utheim T. P. (2015). Postoperative changes in corneal epithelial and stromal thickness profiles after photorefractive keratectomy in treatment of myopia. *Journal of Refractive Surgery*.

[9] Fraunfelder F. W., Wilson S. E. (2001). Laser in situ keratomileusis versus photorefractive keratectomy in the correction of myopic astigmatism. *Cornea*.

[10] Kamiya K., Shimizu K., Ohmoto F. (2009). Comparison of the changes in corneal biomechanical properties after photorefractive keratectomy and laser in situ keratomileusis. *Cornea*.

[11] Fadlallah A., Fahed D., Khalil K. (2011). Transepithelial photorefractive keratectomy: clinical results. *Journal of Cataract and Refractive Surgery*.

[12] Vogt A. (1981). *Textbook and Atlas of Slit Lamp Microscopy of the Living Eye*.

[13] Keane P. A., Bhatti R. A., Brubaker J. W., Liakopoulos S., Sadda S. R., Walsh A. C. (2009). Comparison of clinically relevant findings from high-speed fourier-domain and conventional time-domain optical coherence tomography. *American Journal of Ophthalmology*.

[14] Ma X. J., Wang L., Koch D. D. (2013). Repeatability of corneal epithelial thickness measurements using fourier-domain optical coherence tomography in normal and post-LASIK eyes. *Cornea*.

[15] Ambrósio R., Tervo T., Wilson S. E. (2008). LASIK-associated dry eye and neurotrophic epitheliopathy: pathophysiology and strategies for prevention and treatment. *Journal of Refractive Surgery*.

[16] Wilson S. E. (2001). Laser in situ keratomileusis-induced (presumed) neurotrophic epitheliopathy. *Ophthalmology*.

[17] Tomás-Juan J., Murueta-Goyena Larrañaga A., Hanneken L. (2015). Corneal regeneration after photorefractive keratectomy: a review. *Journal of Optometry*.

[18] Tang M., Li Y., Huang D. (2015). Corneal epithelial remodeling after LASIK measured by fourier-domain optical coherence tomography. *Journal of Ophthalmology*.

[19] Anera R. G., Jiménez J. R., Del Barco L. J., Bermúdez J., Hita E. (2003). Changes in corneal asphericity after laser in situ keratomileusis. *Journal of Cataract and Refractive Surgery*.

[20] Gatinel D., Malet J., Hoang-Xuan T., Azar D. T. (2002). Analysis of customized corneal ablations: theoretical limitations of increasing negative asphericity. *Investigative Ophthalmology and Visual Science*.

[21] Gatinel D., Racine L., Hoang-Xuan T. (2007). Contribution of the corneal epithelium to anterior corneal topography in patients having myopic photorefractive keratectomy. *Journal of Cataract and Refractive Surgery*.

[22] Kwon Y., Bott S. (2009). Postsurgery corneal asphericity and spherical aberration due to ablation efficiency reduction and corneal remodelling in refractive surgeries. *Eye*.

[23] Huang D., Tang M., Shekhar R. (2003). Mathematical model of corneal surface smoothing after laser refractive surgery. *American Journal of Ophthalmology*.

